# Stroke is still a neglected disease in Brazil: Acidente vascular cerebral permanece doença negligenciada no Brasil

**DOI:** 10.1590/1516-3180.2015.13360510

**Published:** 2015-04-14

**Authors:** Paulo Andrade Lotufo

**Affiliations:** I MD, DrPH. Full Professor, Department of Internal Medicine, Faculdade de Medicina da Universidade de São Paulo (FMUSP), São Paulo, Brazil.

Data from the Brazilian Ministry of Health show that the absolute number of deaths due to coronary heart disease surpassed the number of some fatal cerebrovascular events in Brazil only in 2011, for all ages. Higher risk of death due to heart disease than due to cerebrovascular diseases is the pattern in the Western hemisphere. However, as pointed out ten years ago in this *Journal *, Brazil had the highest age-adjusted stroke death rate of all Latin-American countries.^1^ Despite the decline in stroke death rate throughout this country,^2^ the risk of premature death due to stroke in Brazil is one of the highest in the world. 

To prove this statement, we take data from the Global Burden of Disease 2013 study relating to mortality and years of life lost (YLL) due to premature death.^3^ ^4^ First, we compare the years of life lost due to coronary heart disease (CHD) and stroke in South America (except Guyana and Suriname). Second, we compare the YLL of Brazil with that of 18 other selected countries (total of 19 countries). For both comparisons, there is an average value and there are places that can be described as presenting three situations: significantly higher than the mean; indistinguishable from the mean and considerably lower than the mean.

The YLL due to CHD is higher in Venezuela and Paraguay, and Brazil is ranked third, but with values indistinguishable from the mean (Figure 1a). However, the YLL for cerebrovascular diseases is considerably higher in Paraguay and Brazil (Figure 1b).

**Figure 1:**
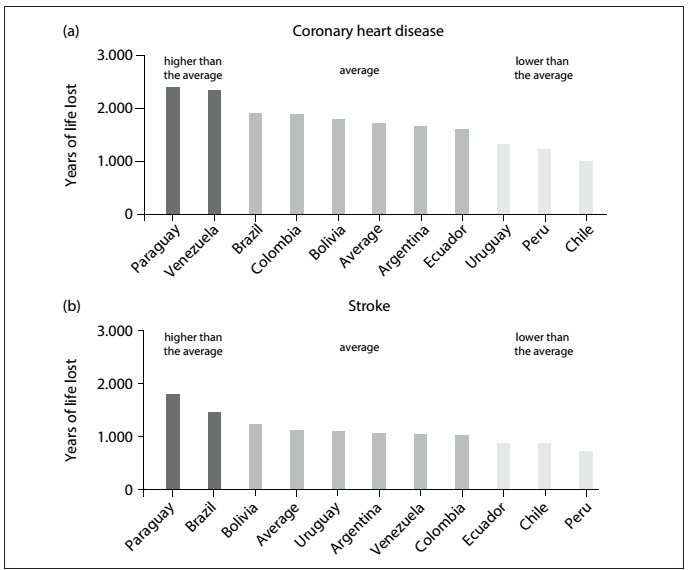
Age-adjusted years of life lost (in thousands) for coronary heart disease (a) and stroke (b) among South American countries in 2013.^4^

Comparison among the selected countries reveals that the YLL due to CHD in Brazil is situated at the mean of the other countries (Figure 2a). On the other hand, for YLL due to cerebrovascular diseases, Brazil is ranked seventh out of 19 countries, with values significantly higher than the mean (Figure 2b).

**Figure 2:**
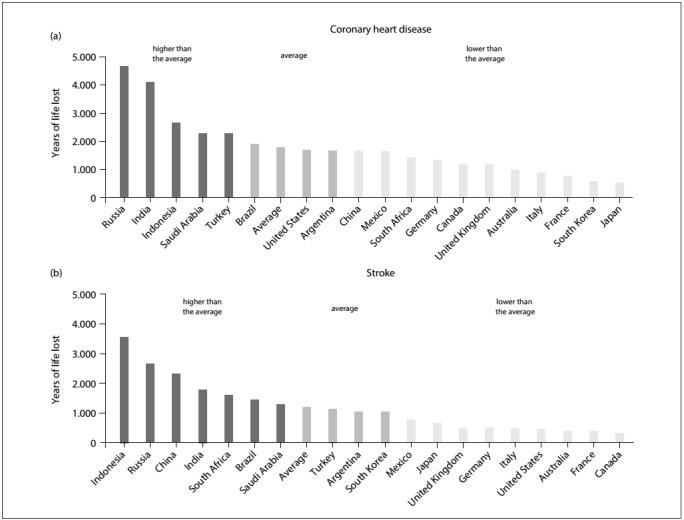
Age-adjusted years of life lost (in thousands) for coronary heart disease (a) and stroke (b) among 19 selected countries in 2013.^4^

The Global Burden of Disease study established an innovative analytical approach towards the official health statistics.^3^ ^4^ However, comparison of health statistics among countries has two main limitations. One is nosological, relating to the coverage of the mortality system and quality of the death certification, which can weaken these comparisons. The other relates to the fact that mortality information systems based on the underlying cause of death have a primary limitation with regard to competing causes of mortality between countries. For example, Bolivia and Ecuador have lower rates of CHD and stroke than Brazil. In contrast, the YLL due to respiratory infections and stomach cancer are significantly higher in Bolivia and Ecuador than in Brazil. Consequently, these Andean countries have lower probability of cardiovascular death than that of Brazil or other South American countries like Argentina.

Apart from these important points, it is undeniable that the burden of stroke is still high in Brazil and that the cardiovascular epidemiological transition in Brazil has been delayed by the burden of stroke mortality.^5^ One important factor in this regard relates to the social, racial and regional differences in stroke mortality in Brazil, in comparison with elsewhere, with high risk among the poorest segments, blacks and people living in northern and northeastern Brazil.^6^ ^7^ ^8^

In relation to these two components of mortality, there is no conclusive data about their incidence in Brazil, but we can speculate that case fatality is declining because of the rise in the numbers of elderly people dying due to stroke sequelae.^2^ Moreover, the Brazilian National Health Survey described point prevalences of self-reported stroke of 1.6% and 1.4%, among men and women, respectively. The prevalences of post-stroke disabilities were 29.5% for men and 21.5% for women.^9^

Recently, an editorial in the journal Arquivos de Neuro-Psiquiatria signed by Fernandes provided a very good summary of the situation of policies for halting the stroke burden in Brazil. Fernandes rightly stated that "we are doing badly due to delays and an inability to implement what is known (cost-effective prevention), causing suffering (morbidity), loss of many lives (mortality) and financial loss." Furthermore, he concluded that "...there has been insufficient investment in evaluating the effects of populational and non-pharmacological interventions, health services for people with CVD are poorly organized...".^10^

The first editorial about stroke as a neglected disease in Brazil was published in 2005. We are hoping to be invited in 2025 to write a narrative with a tentative title of "How Brazil did the right thing towards curbing stroke mortality".

## References

[B1] Lotufo PA (2005). Stroke in Brazil: a neglected disease. Sao Paulo Med J.

[B2] Lotufo PA, Goulart AC, Fernandes TG, Benseñor IM (2013). A reappraisal of stroke mortality trends in Brazil (1979-2009). Int J Stroke.

[B3] GBD 2013 Mortality and Causes of Death Collaborators (2015). Global, regional, and national age-sex specific all-cause and cause-specific mortality for 240 causes of death, 1990-2013: a systematic analysis for the Global Burden of Disease Study 2013. Lancet.

[B4] Murray CJ, Barber RM, GBD 2013 DALYs and HALE Collaborators (2015). Global, regional, and national disability-adjusted life years (DALYs) for 306 diseases and injuries and healthy life expectancy (HALE) for 188 countries, 1990-2013: quantifying the epidemiological transition. Lancet.

[B5] Lotufo PA, Benseñor IM (2009). Stroke mortality in Brazil: one example of delayed epidemiological cardiovascular transition. Int J Stroke.

[B6] Vincens N, Stafström M (2015). Income Inequality, Economic Growth and Stroke Mortality in Brazil: Longitudinal and Regional Analysis 2002-2009. PLoS One.

[B7] Fernandes TG, Bando DH, Alencar AP, Benseñor IM, Lotufo PA (2015). Income inequalities and stroke mortality trends in Sao Paulo, Brazil, 1996-2011. Int J Stroke.

[B8] Lotufo PA, Bensenor IJ (2013). Raça e mortalidade cerebrovascular no Brasil [Race and stroke mortality in Brazil]. Rev Saude Publica.

[B9] Bensenor IM, Goulart AC, Szwarcwald CL, (2015). Prevalence of stroke and associated disability in Brazil: National Health Survey - 2013. Arq Neuropsiquiatr.

[B10] Fernandes JG (2015). Stroke prevention and control in Brazil: missed opportunities. Arq Neuropsiquiatr.

